# Southern Medical Reports

**Published:** 1849-10

**Authors:** 


### SOUTHERN MEDICAL REPORTS.

We owe our friend, Dr. E. D. Fenner, an apology for having so long neglected to call the attention of our readers to a publication which he proposes to commence in New Orleans during the ensuing year. In his prospectus he states that his object in establishing such a work is to "collect and present in a durable form the observations of physicians residing in different parts of the Southern States." It is to be made up of *general* and *special* reports; the first of which will contain the meteorology, medical topography and prevailing diseases throughout the year; the second will be devoted to extraordinary cases, surgical operations, &c.

These reports are to be placed in the hands of Dr. Fenner by the first of January, and will be issued in a neatly bound volume so soon thereafter as the work can be executed. In addition to these original papers, each volume will also contain a brief retrospect of the latest discoveries and improvements contained in the medical journals of the year.

Physicians cannot fail to perceive the value of such an annual summary, and we shall be glad to learn that the encouragement offered has been such as to induce Dr. Fenner to proceed with its publication.

